# Long working hours and all-cause mortality in China: A 26-year follow-up study

**DOI:** 10.5271/sjweh.4115

**Published:** 2023-11-01

**Authors:** Yeen Huang, Yingping Xiang, Wei Zhou, Guanpeng Li, Chengzhi Zhao, Di Zhang, Shenying Fang

**Affiliations:** 1 School of Public Health and Emergency Management, Southern University of Science and Technology, China.; 2 Occupational Hazard Assessment Institute, Shenzhen Prevention and Treatment Center for Occupational Diseases, China.

**Keywords:** death, smoking, China Health and Nutrition Surveys, CHNS

## Abstract

**Objective:**

The relationship between long working hours and the risk of mortality has been debated in various countries. This study aimed to investigate the association between long working hours and all-cause mortality in a large population-based cohort in China.

**Methods:**

This retrospective cohort study (N=10 269) used a large, nationally representative data set [the China Health and Nutrition Surveys (CHNS)] from 1989 to 2015. Long working hours (≥55 hours per week) were compared to standard working hours (35–40 hours per week). The outcome measure was all-cause mortality. Hazard ratio (HR) for all-cause mortality was calculated from Cox proportional hazards regression models, with stratified analyses to assess differences in mortality risk among subgroups.

**Results:**

Among the participants, 411 deaths (3.52 per 1000 person-years) occurred during a median follow-up of 11.0 (range 4.0–18.0) years. After adjusting for covariates, long working hours were associated with a significantly increased risk of all-cause mortality [HR 1.49, 95% confidence intervals (CI) 1.02–2.18]. Stratified analyses revealed that this association was present only among men (HR 1.78, 95% CI 1.15–2.75) and smoking participants (HR 1.57, 95% CI 1.05–2.57).

**Conclusion:**

This study provides evidence of an association between long working hours and all-cause mortality, which is specifically observed among men and smokers. Targeted interventions should be implemented to reduce excessive working hours and identify individuals at elevated risk, with support from labor organizations, policymakers, and employers.

Long working hours, defined as exceeding the standard full-time schedule of typically 35–40 hours per week, have become increasingly prevalent in the labor market (1, 2). However, mounting evidence suggests that this practice may have deleterious effects on health (3). The most extreme and rare consequence is *karoshi*, a Japanese term that refers to sudden death related to overworking (4, 5). Recent systematic reviews and meta-analyses have identified an increased risk of stroke and ischemic heart disease among individuals working ≥55 hours per week compared to those working standard hours (6). As working hours in East Asian countries such as South Korea and Japan are generally longer than those in western countries, death related to overwork, usually from cardiovascular disease (CVD), has become a growing social concern (7, 8).

In China, many sectors have experienced rapid development in recent years, leading to a gradual lengthening of working hours (9). For instance, the internet sector has seen the implementation of the “996” work schedule, requiring employees to work from 09:00–21:00 hours, six days a week. Similar to *karoshi*, a Chinese term, *guolaosi*, has attracted wide attention in Chinese society in recent years. However, the relationship between long working hours and mortality risk remains controversial, with some studies reporting no statistically significant difference (10, 11) while others have linked long working hours to an increased risk of death (12, 13). The controversy may arise from the fact that the mechanism of death caused by long working hours is not yet fully understood. Moreover, most previous cohort studies did not follow up for a sufficient amount of time on death outcomes, resulting in an inability to observe an adequate number of mortality events (14, 15). Long working hours have been linked to an increased risk of CVD (16, 17), which often takes longer to develop. Therefore, insufficient follow-up may lead to an underestimation of the association between long working hours and mortality risk.

The association between long working hours and mortality may be influenced by certain modifying factors, which could potentially impact the magnitude of the relationship. Sex is a well-established determinant of health outcomes, including mortality (18, 19). Prior research has consistently indicated sex-specific differences in health behaviors, disease susceptibility, and response to risk factors (20, 21). In China, men typically shoulder the primary workforce role, facing higher work-related stress than women (22). Additionally, smoking is a known risk factor for major diseases contributing to mortality (23), and China has the highest number of smokers worldwide, with particularly high smoking rates among men (24). Considering these influential factors, our study aimed to explore the impact of sex and smoking status on the association between long working hours and mortality risk. By examining these factors, we aimed to gain valuable insights into the complex interplay among long working hours, sex, smoking, and their combined effects on mortality risk. Understanding these associations is crucial for identifying high-risk populations and developing targeted interventions to mitigate the adverse health consequences of prolonged working hours.

Given that careers consume most of an adult’s time, the impact of long working hours on life expectancy cannot be ignored. The current epidemiological evidence for the association between long working hours and mortality risk primarily stems from studies in South Korea, Japan, and some European countries (4, 5, 10–12, 14, 15). Since China is undergoing rapid economic and social development, it remains unclear whether long working hours would exert similar effects on life expectancy as in East Asian countries with similar sociocultural backgrounds. Moreover, identifying subgroups at high risk of death is crucial for developing appropriate working hours policies and implementing interventions (8).

In this study, we conducted a longitudinal investigation of the association between long working hours and all-cause mortality risk in a Chinese population, utilizing data from a 26-year follow-up study of the China Health and Nutrition Surveys (CHNS). We further performed a stratified analysis by sex and smoking to identify high-risk subgroups of mortality risk.

## Methods

### Study design and sample

This study utilized data from the CHNS, a longitudinal survey conducted by the University of North Carolina at Chapel Hill and Chinese Center for Disease Control and Prevention between 1989 and 2015. The CHNS utilized a multistage, cluster random sampling design to collect data every two to four years in 15 provinces of China, including 12 representative provinces and three centrally-administered municipalities. Informed consent was obtained from all participants and the corresponding institutional review committees approved the study. Detailed information about the survey can be obtained from the official website (www.cpc.unc.edu/projects/china).

**Figure 1:**
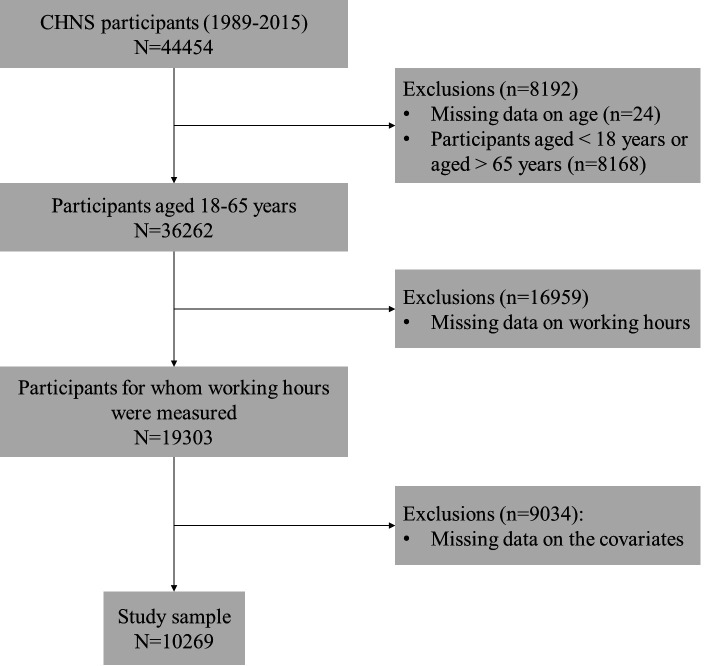


The present study analyzed longitudinal data from the CHNS datasets from 1989 to 2015, including 44 454 participants. Of these, 19 303 were aged 18–65 years and reported working hours and registered all causes of death. Participants who had missing values of covariates were excluded, resulting in a final sample of 10 269 participants. A flowchart of the participant selection process is shown in figure 1. All survey data were self-reported under the supervision of trained investigators using a face-to-face approach, ensuring the accuracy and reliability of the collected information. This study followed the Strengthening the Reporting of Observational Studies in Epidemiology (STROBE) reporting guideline (25). As all data were completely deidentified, consent was waived, and human participant review by the Southern University of Science and Technology institutional review board was not required.

### Working hours

Working hours were self-reported by participants in response to an open-ended question: “How many hours do you usually work per week?” The measurement of working hours was based on the initial survey wave (baseline) when participants were first included in the study (10). Chinese labor law regulations stipulate a standard working hour system of 8 hours per day and 40 hours per week (26, 27). Working hours were categorized into four groups: <35, 35–40, 41–54, and ≥55 hours per week. Standard working hours were defined as working 35–40 hours per week, and long working hours were classified as working ≥55 hours per week, consistent with previous studies (16, 28).

### All-cause mortality

The study endpoint was all-cause death. Participants were followed from the beginning of the calendar year that followed their baseline interview. Follow-up ended at the time they were lost to follow-up, died, or at the end of the study period, whichever occurred first. Date of death was obtained from death registry during each follow-up visit.

### Covariates

Baseline covariates and potential effect modifiers included sex (men or women), age, marital status (unmarried, married, or divorced/separated/widowed), income [classified into high (>75^th^ median), intermediate (25–75^th^ median), or low (<25^th^ median) by interquartile], occupational categories [manual (agricultural workers, unskilled workers, service workers, athletes, and firefighters) or non-manual (professional and technical workers, managers, and office workers)] (29), education level (primary school or less, middle or high school, college or university or above), residence (urban or rural), smoking (no or yes), alcohol consumption (no or yes), and body mass index [BMI, classified as underweight (<18.5 kg/m^2^), normal weight (18.5–23.9 kg/m^2^), overweight (24.0–27.9 kg/m^2^), or obesity (>27.9 kg/m^2^)] (30).

### Statistical analysis

Baseline characteristics of study participants were described according to their working hours. Kaplan-Meier method were constructed to compare survival probabilities of participants with different working hours.

In our survival analyses, the entry date was defined as the date of completion of the baseline survey when studying the effect of baseline characteristics on all-cause mortality. Each participant was observed from the entry date until lost to follow-up, died, or the study period ended, whichever occurred first. Univariate Cox regression models were used to assess the unadjusted impact of working hours and other variables on all-cause mortality. Additional multivariable Cox regression models were adjusted for age, sex, marital status, residence, income, occupational categories, education level, smoking, alcohol consumption, and BMI to examine the independent association between long working hours and all-cause mortality. We verified the assumption of proportional hazards before reporting any results. Hazard ratio (HR), adjusted HR (HR_adj_), and 95% confidence intervals (CI) were reported.

Stratified analyses were carried out by sex and smoking, testing for differences in the association between long working hours and all-cause mortality in different subgroups to identify high-risk groups. We examined the interaction [long working hours × sex (or smoking)] between sex (or smoking) and long working hours on all-cause mortality risk. To determine the presence of a synergistic effect, we calculated the Synergy Index (SI) using the formula: SI=(RR_11_-1)/[(RR_10_-1) + (RR_01_-1)], where RR_11_ represents the relative risk (RR) for individuals exposed to long working hours and sex (or smoking), RR_10_ represents the RR for individuals exposed to sex (or smoking) only, and RR_01_ represents the RR for individuals exposed to long working hours only. To examine the potential public health importance of long working hours as a risk factor for all-cause mortality, we computed population attributable fractions (PAF) using prevalence estimates for participants with long working hours and HR_adj_. PAF provides an estimate of the proportion of death that could be avoided in the population if exposure to long working hours was completely removed assuming that the association between long working hours and mortality is causal (31). PAF was calculated as PAF=*p*(HR-1)/[1+*p*(HR-1)], in which *p* is the frequency of long working hours in the total population at baseline and HR is the HR for all-cause mortality for long versus standard working hours.

All P-value were from two-sided tests, and results were deemed statistically significant at P <0.05. Statistical analyses were performed using IBM Statistics SPSS version 26.0 (IBM Corp, Armonk, NY, USA).

## Results

### Baseline characteristics of participants by working hours

In this study, 10 269 participants were enrolled, of whom 24.0% reported working long hours (≥55 hours/week). The median age of the participants was 49.0 (interquartile range 42.0–58.0) years, with 52.9% men, most married, over half middle-income earners, and over one-third smokers. The baseline characteristics of participants grouped by working hours are shown in table 1.

**Table 1:** Baseline characteristics of participants by working hours, N=10 269.

Variables ^a^		All (N=10 269)		Working hours (hours/week)						
				<35 (N=1931)		35–40 (N=3154)		41–54 (N=2718)		≥55 (N=2466)
		N (%)		N (%)		N (%)		N (%)		N (%)
Sex										
	Men	5432 (52.9)		846 (43.8)		1655 (52.5)		1514 (55.7)		1417 (57.5)
	Women	4837 (47.1)		1085 (56.2)		1499 (47.5)		1204 (44.3)		1049 (42.5)
Marital status										
	Unmarried	1394 (13.6)		160 (8.3)		358 (11.4)		495 (18.2)		381 (15.5)
	Married	8631 (84.0)		1717 (88.9)		2712 (86.0)		2178 (80.1)		2024 (82.0)
	Divorced/separated/widowed	244 (2.4)		54 (2.8)		84 (2.6)		45 (1.7)		61 (2.5)
Income										
	Low	1373 (13.4)		458 (23.7)		95 (3.0)		427 (15.7)		393 (15.9)
	Intermediate	5571 (54.2)		1171 (60.7)		1293 (41.0)		1672 (61.5)		1435 (58.2)
	High	3325 (32.4)		302 (15.6)		1766 (56.0)		619 (22.8)		638 (25.9)
Occupational categories										
	Non-manual	3089 (30.1)		166 (8.6)		1879 (59.6)		740 (27.2)		304 (12.3)
	Manual	7180 (69.9)		1765 (91.4)		1275 (40.4)		1978 (72.8)		2162 (87.7)
Education level										
	Primary school or less	3230 (31.5)		1082 (56.0)		352 (11.2)		837 (30.8)		959 (38.9)
	Middle or high school	5582 (54.4)		786 (40.7)		1769 (56.0)		1626 (59.8)		1401 (56.8)
	College or university or above	1457 (14.1)		63 (3.3)		1033 (32.8)		255 (9.4)		106 (4.3)
Residence										
	Urban	4363 (42.5)		345 (17.9)		1926 (61.1)		1323 (48.7)		769 (31.2)
	Rural	5906 (57.5)		1586 (82.1)		1228 (38.9)		1395 (51.3)		1697 (68.8)
Smoking										
	No	6714 (65.4)		1320 (68.4)		2148 (68.1)		1734 (63.8)		1512 (61.3)
	Yes	3555 (34.6)		611 (31.6)		1006 (31.9)		984 (36.2)		954 (38.7)
Alcohol consumption										
	No	6071 (59.1)		1307 (67.7)		1817 (57.6)		1539 (56.6)		1408 (57.1)
	Yes	4198 (40.9)		624 (32.3)		1337 (42.4)		1179 (43.4)		1058 (42.9)
Body mass index										
	Underweight	616 (6.0)		141 (7.3)		145 (4.6)		178 (6.5)		152 (6.2)
	Normal	6565 (63.9)		1292 (66.9)		1862 (59.1)		1802 (66.3)		1609 (65.2)
	Overweight	2382 (23.2)		403 (20.9)		881 (27.9)		575 (21.2)		523 (21.2)
	Obese	706 (6.9)		95 (4.9)		266 (8.4)		163 (6.0)		182 (7.4)

^a^ Median age [interquartile range (IQR)] for all participants was 49.0 (42.0–58.0) years. For those working <35, 35–40, 41–54, and ≥55 hours/week, median ages (IQR) were 52.0 (43.0–59.0), 47.0 (40.0–55.0), 51.0 (44.0–58.0), and 48.0 (42.0–55.0) years, respectively.

### Comparison of all-cause mortality in different working hours

During the study period, the median time to death or censoring was 11.0 (range 4.0–18.0) years, with a follow-up time of 116 705 person-years among all participants (supplementary material, www.sjweh.fi/article/4115, table S1). As illustrated in supplementary table S2, a total of 411 deaths (4.0%) occurred among all participants. The all-cause mortality rate for participants working ≥55 hours per week was 4.15 per 1000 person years, compared to 1.67 person years for those in the standard working hours group. The log-rank test demonstrated significant differences in survival rates among different working hours groups (χ^2^=54.11, P<0.001).

### Associations between long working hours and all-cause mortality risk

Univariate Cox regression analysis showed that long working hours were significantly associated with an increased all-cause mortality risk (HR 2.39, 95% CI 1.69–3.38, supplementary table S3). After adjusting for the effects of covariates, multivariable Cox regression analysis demonstrated that long working hours were independently associated with a higher risk of all-cause mortality compared to working standard hours (HR_adj_ 1.49, 95% CI 1.02–2.18, supplementary table S3). The population attributable fraction (PAF) of long working hours for all-cause mortality in the total population was 10.52% (c).

### Associations between long working hours and all-cause mortality risk stratified by sex and smoking

We included interaction terms for long working hours and sex, as well as long working hours and smoking, in our models. The results indicated significant interaction effects between long working hours and sex (P=0.026), as well as between long working hours and smoking (P=0.039). Therefore, we conducted a stratified analysis to investigate the modifying effect of sex or smoking on the association between long working hours and all-cause mortality risk. Stratification by sex (table 2a) or smoking (table 2b) revealed that the association between long working hours and all-cause mortality remained statistically significant only among men (HR_adj_1.78, 95% CI 1.15–2.75) and smokers (HR_adj_ 1.57, 95% CI 1.05–2.57). Univariate hierarchical Cox regression results were presented in supplementary table S4. The synergy index for long working hours and sex on mortality was 0.50, and for long working hours and smoking was 0.44. When stratified by both sex and smoking (table 4), an association between long working hours and all-cause mortality risk was observed only among male smokers (HR_adj_ 1.72, 95% CI 1.03–2.87). The PAF of long working hours for all-cause mortality among men, smokers, and male smokers was 16.91%, 13.25%, and 16.17%, respectively (table 3).

**Table 2a:** Association between long working hours and all-cause mortality risk stratified by **sex** (N=10 269). [HR_adj_=adjusted hazard ratio; CI=confidence intervals.]

Variables		Women				Men		
		Deaths/Exposed (N)	HR_adj_^a^	95% CI		Deaths/Exposed (N)	HR_adj_^a^	95% CI
Working hours (hours/week)								
	<35	35/1085	1.03	0.59–1.58		64/846	1.46	0.89–2.36
	35–40	13/1499	1.00	–		32/1655	1.00	–
	41–54	22/1204	0.68	0.31–1.47		126/1514	1.51	0.99–2.30
	≥55	15/1049	0.67	0.29–1.55		104/1417	1.78	1.15–2.75 ^b^

^a^ Adjusted for age, marital status, income, occupational categories, education level, residence, smoking, alcohol consumption, and body mass index (BMI). Significant interaction (long working hours × sex) by sex in the association between long working hours and all-cause mortality risk (P=0.026). Synergy index (long working hours × sex) =0.50. ^b^ P<0.05.

**Table 2b:** Association between long working hours and all-cause mortality risk stratified by **smoking** (N=10 269). [HR_adj_=adjusted hazard ratio; CI=confidence intervals.]

Variables		Non-smoker				Smoker		
		Deaths/Exposed (N)	HR_adj_^a^	95% CI		Deaths/Exposed (N)	HR_adj_^a^	95% CI
Working hours (hours/week)								
	<35	47/1320	1.58	0.86–2.92		52/611	1.41	0.82–2.42
	35–40	19/2148	1.00	–		26/1006	1.00	–
	41–54	56/1734	1.26	0.71–2.24		92/984	1.30	0.81–2.10
	≥55	39/1512	1.36	0.75–2.50		80/954	1.57	1.05–2.57 ^b^
^a^ Adjusted for age, sex, marital status, income, occupational categories, education level, residence, alcohol consumption, and body mass index (BMI). Significant interaction (long working hours × smoking) by smoking in the association between long working hours and all-cause mortality risk (P=0.039). Synergy index (long working hours × smoking) =0.44. ^b^ P<0.05.								

**Table 3:** Population attributable fraction for long working hours versus standard working hours in different groups of participants. Hazard ratios for the calculation of PAF were adjusted for covariates. [PAF= population attributable fraction; CI=confidence intervals.]

Variables	% (95% CI)
PAF for total population	10.52 (0.48–22.07)
PAF for men	16.91 (3.77–31.35)
PAF for smokers	13.25 (1.32–29.62)
PAF for male smokers	16.17 (0.80–33.38)

**Table 4:** Association between long working hours and all-cause mortality risk stratified by sex and smoking, N=5432. [HR_adj_=adjusted hazard ratio; CI=confidence intervals.]

Variables		Male non-smokers						Male smokers				
		Deaths/Exposed (N)	HR	95% CI	HR_adj_^a^	95% CI		Deaths/Exposed (N)	HR	95% CI	HR_adj_^a^	95% CI
Working hours (hours/week)												
	<35	13/280	2.68	1.52–6.88 ^b^	1.22	0.45–3.31		51/566	2.54	1.18–3.76 ^c^	1.56	0.89–2.73
	35–40	8/669	1.00	–	1.00	–		24/986	1.00	–	1.00	–
	41–54	37/558	2.86	1.32–6.22 ^b^	1.76	0.79–3.93		89/956	1.84	1.16–2.90 ^c^	1.42	0.87–2.33
	≥55	25/499	2.94	1.32–6.54 ^b^	2.01	0.87–4.65		79/918	2.52	1.59–3.98 ^c^	1.72	1.03–2.87 ^d^

^a^ Adjusted for age, marital status, income, occupational categories, education level, residence, alcohol consumption, and body mass index. ^b^ P<0.01. ^c^ P<0.001. ^d^ P<0.05.

We performed sensitivity analyses to address exposure misclassification due to change in working hours over time. With time-dependent exposure to long working hours, the main findings were replicated (supplementary table S5~S6).

## Discussion

In this 26-year follow-up study, we investigated the association between long working hours and all-cause mortality risk among Chinese workers aged 18–65 years. Our results suggest that long working hours are associated with an increased risk of all-cause mortality, with a population attributable fraction of 10.52%. This finding underscores the need to address the death burden caused by long working hours in China. Stratified analyses revealed that the association between long working hours and mortality risk was only observed among men and smokers. Notably, the association remained significant among male smokers. This observation indicates that interventions aimed at reducing long working hours should focus on specific subgroup to mitigate the adverse health outcomes associated with long working hours.

To the best of our knowledge, our study is the first population-based cohort study to examine the association between working hours and mortality risk in a Chinese population. Our findings are consistent with previous research in South Korea and Japan, which reported a positive association between longer working hours and mortality risk (4, 5, 8, 12). However, in contrast to some European countries, which have tended to report no association (10) or even a negative association with mortality risk (14), our study highlights regional variations in the association between long working hours and mortality risk. These reginal variations in findings may be influenced by cultural attitudes towards work and work-life balance, work-related policies and practices, and differences in study populations. In East Asian cultures, working long hours is often viewed as a sign of dedication and commitment, resulting in a higher prevalence of long working hours and an increased risk of adverse health outcomes (32, 33). In contrast, some European countries have regulations that limit working hours and promote work-life balance (34, 35). These differences in policies and practices may contribute to the regional variations in findings.

Our study contributes to the growing body of evidence linking long working hours with increased mortality risk, specifically among men and smokers. These findings highlight the importance of targeted prevention strategies and interventions to mitigate the negative health consequences of long working hours in these high-risk group. The observed association between long working hours and mortality risk can be attributed to various factors, including occupational stress, unhealthy behaviors, and biological mechanisms such as inflammation and metabolic disorders (36, 37). Studies on Karoshi, a phenomenon associated with death from overwork, suggest that work-related stress may lead to the secretion of catecholamines (epinephrine and norepinephrine) and cortisol, which can contribute to the development of atherosclerosis and an increased risk of CVD and stroke (38). While the exact pathophysiological mechanisms by which occupational stress induces and exacerbates CVD and stroke are not yet fully understood, research suggests that overwork may accelerate the thrombotic reactions through the hypothalamic-pituitary-adrenal (HPA) axis and the sympathetic nervous system (39).

In addition to stress-related factors, attention should also be given to unhealthy behaviors in the workplace. A case–control study conducted in China demonstrated that prolonged working hours in sedentary occupations were associated with an increased risk of coronary heart disease, even after controlling for the effects of physical activity during leisure times (40). Another study among Chinese men found that working >60 hours per week and having <6 hours of sleep per day significantly increased the risk of CVD, even after accounting for factors such as smoking and psychosocial work-related factors (41). Disruptions of circadian rhythms, sleep patterns, and increased exposure to environmental stressors, including air pollution, may further contribute to the elevated risk of mortality associated with long working hours (39, 42, 43). However, further research is needed to fully elucidate the complex biological mechanisms underlying *karoshi* and to control for potential confounding factors and comorbidities that may influence the accuracy of effect estimates.

Our study suggests several prevention strategies and interventions to alleviate the negative health consequences of long working hours among occupational populations. Firstly, routine health screenings and counseling services provided by healthcare providers can help to identify and manage health risks associated with long working hours. Secondly, occupational health and safety policies should promote work–life balance by limiting working hours (44, 45), especially for men or smokers who face a higher mortality risk. Thirdly, targeted interventions such as smoking cessation programs and tobacco tax increases should aim to reduce smoking prevalence among male workers. Fourthly, promoting regular exercise and healthy eating can mitigate the negative health effects of long working hours and reduce the risk of chronic diseases, such as obesity, diabetes, and CVD (46–48). Finally, workplace policies that allow for breaks and rest periods during long workdays can reduce stress and fatigue, thus improving overall well-being.

The present study has both strengths and limitations. The use of data from a nationally representative cohort, with a long follow-up period of 26 years, provided ample observation time for mortality outcomes. Additionally, this is the first study to investigate the association between long working hours and mortality in China, with a relatively large number of mortality events and narrower 95% CI estimates than other studies, indicating reliable results. However, self-reported data may have introduced reporting bias, and the lack of definitive data on the causes of death prevented analysis of the association between long working hours and the risk of death from specific diseases. Additionally, due to the use of public databases in our research, it is inevitable that certain variables may have missing values, which could potentially lead to a biased estimate of our results. Furthermore, despite the longer follow-up duration in our study compared to other studies, the number of all-cause mortality events observed was relatively small compared to studies focusing on specific diseases such as anxiety, depression, and alcohol use (49, 50). Since long working hours are a prevalent occupational risk factor with the largest attributable disease burden (51), future prospective cohort studies with larger samples are needed to further identify the long-term effects and mechanisms of long working hours on health outcomes. Finally, potential confounders, such as shift work, seasonal variations, and biomarkers, were not measured or analyzed in this study, which could have influenced the results. Despite these limitations, our study contributes to the growing body of evidence indicating the adverse health effects of long working hours and underscores the importance of implementing policy interventions and workplace health promotion programs specifically designed for high-risk populations. The measures are crucial in mitigating the associated risks and promoting the well-being of workers.

### Concluding remarks

In conclusion, our study provides evidence that long working hours are associated with an increased risk of all-cause mortality, which is specifically observed among men and smokers. These findings emphasize the need for interventions aimed at reducing excessive working hours and promoting work–life balance, as well as targeted interventions for high-risk group. Concerted efforts by labor organizations, policymakers, and employers are essential to address this important public health issue and improve the health and well-being of workers. Future research is needed to identify the underlying mechanisms and specific diseases associated with long working hours and to evaluate the effectiveness of interventions aimed at reducing the negative health effects of long working hours.

### Ethics approval

As all data were completely deidentified, this study did not require human participants review by the Southern University of Science and Technology institutional review board. Consent was waived because all data were deidentified.

### Additional information

The CHNS data are publicly available at www.cpc.unc.edu/projects/china/data/datasets/index.html. Dissemination to study participants is not possible/applicable given the nature of public use and deidentified CHNS data.

## Supplementary material

Supplementary material
