# Supplementary material for: Long working hours and all-cause mortality in China: A 26-year follow-up study
Source: Scand J Work Environ Health. 2023 Oct 31;49(8):539–48. doi: 10.5271/sjweh.4115 (PMC10857867; doi:10.5271/sjweh.4115)
Supplement: Supplementary material [file SJWEH-49-539-S001.pdf]

Long working hours and all-cause mortality in China: A 26-year follow-up study[1]

by Yeen Huang, MD, Yingping Xiang, MM, Wei Zhou, MM, Guanpeng Li, MD, Chengzhi Zhao, MM, Di Zhang, MM, Shenying Fang, MD, PhD [2]

1. Supplementary material
2. Correspondence to: Shenying Fang, MD, PhD, Professor, School of Public Health and Emergency Management, Southern University of Science and Technology, No. 1088, Xueyuan Avenue, Nanshan District, Shenzhen, Guangdong Province, China. [E-mail: fangsy@sustech.edu.cn]

Supplemental Table S1. Results of participants' follow-up time, N=10269.

Variables	Follow-up time (year)
**Mean±SD**	11.4±8.2
**Median (Interquartile)**	11.0 (4.0-18.0)
**Total person-years**	116705

Supplemental Table S2. All-cause mortality (per 1000 person-years) in different working hours during follow-up, N=10269.

Variables	Person-years (PY)<br>N	Numbers of deaths<br>N	Numbers of exposed<br>N	Deaths per 1000 PY<br>N	Deaths per exposed<br>%	χ^2	*P*-value of Log-rank test[a]
**All**	116705	411	10269	3.52	4.0	-	-
**Working hours (hours/week)**						54.11	<0.001
<35	17864	99	1931	5.54	5.1		
35-40	26935	45	3154	1.67	1.4		
41-54	43259	148	2718	3.42	5.4		
≥55	28647	119	2466	4.15	4.8		

[a]Log-rank test was used to compare the survival rates of different working time groups.

Supplemental Table S3. Association between long working hours and all-cause mortality risk, N=10269. [HR, hazard ratio; CI, confidence intervals; BMI, body mass index.]

Variables	HR	95% CI	Adjusted HR	95% CI
**Working hours (hours/week)**				
<35	**3.39**	**2.38-4.83***	1.47	0.98-2.21
35-40	1.00	-	1.00	-
41-54	**1.86**	**1.32-2.61***	1.29	0.90-1.87
≥55	**2.39**	**1.69-3.38***	**1.49**	**1.02-2.18***
**Sex**				
Women	1.00	-	1.00	-
Men	**2.95**	**2.33-3.75***	**2.42**	**1.76-3.33***
**Age**	**1.04**	**1.03-1.04***	**1.02**	**1.01-1.03***
**Marital status**				
Unmarried	1.00	-	1.00	-
Married	**3.30**	**2.25-4.86***	**3.54**	**2.22-5.65***
Divorced/separated/widowed	**6.16**	**3.33-11.40***	**7.01**	**3.51-14.02***
**Income**				
Low	1.00	-	1.00	-
Intermediate	**0.19**	**0.10-0.33***	**0.26**	**0.14-0.49***
High	**0.69**	**0.56-0.87**	**0.71**	**0.55-0.90**
**Occupational categories**				
Non-manual	1.00	-	1.00	-
Manual	**2.22**	**1.69-2.92***	**1.77**	**1.31-2.39***
**Education level**				
Primary school or less	1.00	-	1.00	-
Middle or high school	**0.23**	**0.18-0.28***	**0.32**	**0.24-0.41***
College or university or above	**0.13**	**0.07-0.27***	**0.24**	**0.11-0.54***
**Residence**				
Urban	1.00	-	1.00	-
Rural	**1.84**	**1.48-2.28***	**1.48**	**1.17-1.87**
**Smoking**				
No	1.00	-	1.00	-
Yes	**2.63**	**2.16-3.21***	**1.34**	**1.05-1.73***
**Alcohol consumption**				
No	1.00	-	1.00	-
Yes	**1.88**	**1.55-2.29***	1.20	0.95-1.52
**BMI**				
Underweight	**2.02**	**1.51-2.72***	**2.05**	**1.52-2.75***
Normal	1.00	-	1.00	-
Overweight	0.91	0.70-1.18	0.97	0.74-1.27
Obese	0.93	0.58-1.49	1.01	0.63-1.62

***P<0.001, **P<0.01, *P<0.05.

Supplemental Table S4. Univariate cox regression analysis stratified by sex or smoking, N=10269. [HR, hazard ratio; CI, confidence intervals.]

Variables	Women		Men	
	HR	95% CI	HR	95% CI
**Working hours (hours/week)**				
<35	1.09	0.63-1.86	**1.74**	**1.44-3.72**[***]
35-40	1.00	-	1.00	-
41-54	1.07	0.53-2.18	**2.10**	**1.42-3.12**[***]
≥55	1.16	0.55-2.44	**2.68**	**1.80-4.00**[***]
	Non-smoker		Smoker	
	HR	95% CI	HR	95% CI
**Working hours (hours/week)**				
<35	**1.95**	**1.11-3.14**[*]	**2.13**	**1.95-3.02**[**]
35-40	1.00	-	1.00	-
41-54	**1.90**	**1.12-3.23**[*]	**1.73**	**1.11-2.69**[*]
≥55	**2.07**	**1.19-3.59**[*]	**2.32**	**1.49-3.62**[***]

[***]$P<0.001$, [**]$P<0.01$, [*]$P<0.05$.

Supplemental Table S5. Association between time-dependent long working hours and all-cause mortality risk, N=10269. [HR, hazard ratio; CI, confidence intervals.]

Variables	Adjusted HR[a]	95% CI
**Working hours (hours/week)**		
<35	1.46	0.97-2.29
35-40	1.00	-
41-54	1.29	0.89-1.88
≥55	**1.47**	**1.05-2.29**[*]

[a]Adjusted for age, sex, marital status, income, occupational categories, education level, residence, smoking, alcohol consumption and body mass index (BMI).

[*]$P<0.05$.

Supplemental Table S6. Association between time-dependent long working hours and all-cause mortality risk stratified by sex or smoking, N=10269. [HR, hazard ratio; CI, confidence intervals.]

Variables	Women		Men	
	Adjusted HR[a]	95% CI	Adjusted HR[a]	95% CI
**Working hours (hours/week)**				
<35	1.83	0.84-4.00	1.57	0.95-2.61
35-40	1.00	-	1.00	-
41-54	0.59	0.27-1.29	**1.58**	**1.03-2.42**[*]
≥55	0.40	0.14-1.11	**2.03**	**1.25-3.31**[**]
	Non-smoker		Smoker	
	Adjusted HR[b]	95% CI	Adjusted HR[b]	95% CI
**Working hours (hours/week)**				
<35	2.09	0.96-2.94	1.54	0.87-2.72
35-40	1.00	-	1.00	-
41-54	1.19	0.66-2.12	1.36	0.84-2.21
≥55	1.11	0.56-2.22	**1.79**	**1.03-3.12**[*]

[a]Adjusted for age, marital status, income, occupational categories, education level, residence, smoking, alcohol consumption and body mass index (BMI).

[b]Adjusted for age, sex, marital status, income, occupational categories, education level, residence, alcohol consumption and body mass index (BMI).

[**]$P<0.01$, [*]$P<0.05$.
